# Systems change to improve tobacco use identification and referral in the chiropractic setting: a pilot study

**DOI:** 10.1186/s12998-018-0214-y

**Published:** 2018-11-26

**Authors:** Kelly Buettner-Schmidt, Brody Maack, Mary Larson, Megan Orr, Donald R. Miller, Katelyn Mills

**Affiliations:** 1 College of Health Professions, School of Nursing, North Dakota State University, 1919 University Drive North, D102, Fargo, ND 58102 USA; 2 College of Health Professions, School of Pharmacy, North Dakota State University, Fargo, ND 58102 USA; 3 College of Health Professions, Department of Public Health, North Dakota State University, Fargo, ND 58102 USA; 4 Department of Statistics, North Dakota State University, Fargo, ND 58102 USA; 5 Roger Maris Cancer Center, Sanford Health, Fargo, ND 58102 USA

**Keywords:** Chiropractic, Systems change, Tobacco, Tobacco use disorder, Smoking, Tobacco cessation, Smoking cessation, Public health

## Abstract

**Background:**

Tobacco use remains a leading cause of death and disability in the United States. Health professionals need to address the use of tobacco products by their patients, but chiropractic clinical systems often remain unsupported and underappreciated in their role to facilitate tobacco use cessation.

**Methods:**

This pilot study tested an intervention to assist a chiropractic community to implement sustainable health systems changes for tobacco use based on U.S. Public Health Service guidelines. Chiropractors were educated on the *Ask*, *Advise*, *Refer* (AAR) approach, provided with ongoing guidance, and followed for six months to assess systems change. The study was conducted from March 2016 to July 2017.

**Results:**

Evidence of a systematic process in place to conduct AAR was present in all clinics by the end of the fourth month of the intervention period. Although no clinic had sustained health system change for full AAR, all six of the clinics made progress in the individual AAR components. Furthermore, five clinics achieved sustained system change for the *Ask* component, as after systems change was achieved, the rate of tobacco user identifications did not drop below 50%. For the *Advise* component, five clinics succeeded in having individual months of ≥50% of tobacco users being advised, and three clinics achieved the formal definition of systems change. For the *Refer* component, no clinic achieved system change, although four had individual months of ≥50% of tobacco users being referred. The patient quit rate was 13.3% (*n* = 15) for the 30-day follow-up and 16.7% (*n* = 6) for the three-month follow-up.

**Conclusions:**

This study demonstrates the feasibility of implementing a health systems change in the chiropractic setting to identify tobacco users, to advise them to quit, and to refer users for cessation services.

## Background

Despite the steady decline in the prevalence of tobacco use, more than one in seven adults continue to use tobacco products, and tobacco-related illness remains the leading cause of preventable death in the United States [1]. Coordinated and systematic efforts to implement evidence-based tobacco control and cessation continue to undergo quality improvement [2–5]. Efforts to fully integrate tobacco dependence treatment within health systems are aimed at creating workflows to assess tobacco use, provide advice to quit, and arrange help with quitting through a referral process [2, 3, 6]. Most adults who use tobacco report the desire to quit; however, only slightly more than half have been advised by a health professional to quit and use of counselling and evidence-based interventions to quit is underutilized [6]. Often, health care professionals who do not have systematic reminders to assess, advise, and refer miss opportunities to address tobacco use with their patients [3]. Therefore, systems change that prompts health care professionals to address tobacco use during each patient encounter, along with technical support and assistance such as academic detailing (AD), are important steps toward making tobacco use intervention the standard of care [3]. AD involves delivering education by trained personnel to providers at their practice, and is described later in the paper.

The U.S. Public Health Service provided evidence-based guidelines and support for health systems change to implement tobacco dependence treatment, including opportunities for clinician education [2]. Most health systems change has been implemented within conventional health care settings, including outpatient and inpatient health care organizations. Unfortunately, complementary and alternative medical systems and their clinicians were not included in the rollout of the guidelines. Yet, many adults utilize complementary and alternative medical therapies, some as an adjunct to conventional medicine and others as their primary source of health care. Chiropractic care comprises the largest provision of complementary health care in the United States [7].

Chiropractic colleges across the United States are required to integrate health and wellness competencies within the curriculum [8]. One component of these new competencies includes guidelines for chiropractors to provide a brief intervention using the 5-A’s—Ask, Advise, Assess, Assist, and Arrange—as outlined by the U.S. Surgeon General, for every patient who reported using tobacco products [8]. The Council on Chiropractic Education recognized a need to train chiropractors in health behavior change, although some chiropractors may not want to serve in this capacity [9].

A 2004 survey of faculty and students at 10 U.S. chiropractic colleges found positive attitudes toward providing preventive services, but chiropractic training was not necessarily meeting the needs of practitioners in health promotion and health risk assessment [10]. Also, a 2012 survey of chiropractors in the United Kingdom found that approximately 60% of chiropractors evaluated their patients for smoking as a lifestyle issue, and 58% provided advice on change, but only 20% felt responsible for helping their patients set goals for change [11].

Thus, although some work is being conducted with chiropractors to systematically address patients’ tobacco use [12–15], it is likely that chiropractic clinical systems are unsupported and underappreciated in their role to facilitate tobacco use cessation.

## Methods

### Study aims, design, and setting

The aim of this pilot study was to develop an evidence-based intervention to assist the chiropractic community to implement sustainable health systems changes addressing tobacco use and cessation in their patient population based on the U.S. Public Health Service’s *Clinical Practice Guideline: Treating Tobacco Use and Dependence: 2008 Update* (The Guidelines) [2]*.* Two of the study’s aims were to:

1. Create health systems change based on the integration of The Guidelines and current scientific literature into chiropractor clinics in eastern North Dakota (ND).
2. Demonstrate that chiropractic health systems change can improve tobacco cessation in terms of quit attempts and successful quitting.

The study used an adaptive method that allows for modifications to the design or statistical procedures during its conduct [16]. The study was conducted from March 2016 to July 2017. The setting was chiropractic clinics located within approximately 120 miles of Fargo, ND, and located in ND. Participants included practicing licensed chiropractors seeing English-speaking patients at least 18 years of age. The North Dakota State Board of Chiropractic Examiners (NDSBCE) provided the names of all licensed chiropractors practicing in ND. Of those provided by the NDSBCE, 175 had office addresses within 120 miles of Fargo. Exclusion criteria included any chiropractic practices in a large health care system or that already met the definition of successful health systems change (defined later).

For recruitment, the study’s population (*n* = 175) was mailed a brief survey that included an opportunity for the chiropractors to express an interest in participating in the study. With a response rate of 33.7% (*n* = 59), 3 self-identified as practicing in a large health system and 17 (28.8%) indicated they were interested in participating in the study. Of the 17 interested, 16 met the study criteria, as one was from a large healthcare system. After the 16 received more detailed information about the study, 10 withdrew their initial interest, leaving 6 participating chiropractors. Additionally, study staff gave presentations at three regional chiropractic meetings and attended the North Dakota Chiropractic Association Annual Meeting to recruit chiropractors. Finally, a Chiropractic Advisory Board was formed to provide input into development of the interventions and to encourage participation.

The patients targeted in this study were those whose chiropractic visit was classified as a new episode of care (NEC). Conceptually, chiropractic care involves a systematic pattern of several visits over a short time for one problem, as opposed to a single treatment [17]. Operationally, for this study, the chiropractor providing care to the patients determined when a visit was an NEC.

The definition of health systems change was based on the studies by Land et al. [18] and Moody-Thomas et al. [19]. Health systems change was determined when a clinic met the following definition: the first month when at least half or more of the office visits at a given site documented 1) an identification of tobacco use at the first visit for every NEC, 2) NEC patients who were tobacco users were advised to quit, and 3) NEC patients who used tobacco and were interested were referred for smoking cessation. Furthermore, there must have been at least two consecutive months with rates above 50% in which these activities occurred. Sustained change meant that in all months following, the rate of tobacco user identifications did not drop below 50%.

### Description of process and interventions

#### Education

Participants attended 12 h of education, presented in the evenings and on weekends, using face-to-face and web-based learning, to accommodate the chiropractors’ requests to receive the learning outside of their regular scheduled clinic hours. The sessions began with a presentation by a chiropractor on the opportunities for chiropractor engagement in evidenced-based, mainstream, tobacco control in their clinics by adopting health systems changes to address tobacco use by their patients and future possibilities for reimbursement of clinical time. More formal education included information on the epidemiology and health effects of tobacco; principles of addiction; best practices for tobacco control; the *Ask*, *Advise, Refer* (AAR) brief intervention method [2, 3, 20, 21]; and tobacco cessation treatment. Active learning occurred through sessions on creating health systems change in clinics, motivational interviewing (MI) techniques, and use of a standardized patient scenario (SPS). Because one aspect of creating health systems change in clinics is to assess the current system, the participants used worksheets adapted from the American Academy of Family Physicians [22] to evaluate their current health system and patient flow. Next they created plans for a new patient flow, for standardizing the new system, and developed an implementation plan with deadlines. The participants were encouraged to share their plans with each other. Free resources for tobacco cessation posters, patient handouts, and quitline referrals, along with information on how to order these items in the future, were provided. Quitlines are free telephone smoking cessation services available in all states, sponsored by the individual states in cooperation with the U.S. Department of Health and Human Services, that are staffed by counselors trained in smoking cessation. Participants were awarded 12 h of continuing education by the NDSBCE.

#### Preparation for systems change through academic detailing

AD is recommended by the Centers for Disease Control and Prevention to facilitate systems change. It is defined as “structured visits by trained personnel to health care practices for the purpose of delivering tailored training and technical assistance to health care providers to help them use best practices” ([23] p 1). AD, as an extension of the initial education, was provided by “outreach specialists” (OS) and occurred one to three weeks after the initial education was completed. OS staff received 37 h of extensive training, covering specific topics to be discussed during AD sessions, including AAR processes and systems changes, MI, nicotine addiction, and evidence-based treatment of nicotine addiction, 24 h of which was formal MI training.

Chiropractic clinic staff received training during the first OS AD visit to the clinic, with subsequent sessions provided as needed. AD detailed lesson plans were used by OS staff and included technical support for implementation of the intervention, conduct of the SPS, educational materials with recommended placement, education for reordering, assistance with customization of clinic health records with AAR model language, and identification and training of a clinic site champion as the primary resource for the intervention. The OS answered questions and performed troubleshooting for any difficulties. Ongoing AD occurred in the chiropractors’ offices or by phone support to clinics through the period of the intervention.

OS staff collected data to capture the effectiveness of chiropractor staff implementation of the intervention during the SPS activity. The primary outcome was the appropriate implementation of each component of the AAR process. Additional outcomes included the chiropractor-patient engagement to increase patient comfort and trust and assessment of six supportive behaviors to promote the AAR process, including being nonjudgmental, listening carefully, explaining that tobacco directly affects the patient’s health, addressing the patient’s concerns, offering clear advice, and offering printed materials. This assessment process was adapted from Hawk et al. [24]. Frequency was calculated for AAR, and range and median were reported for each supportive behavioral item on a six-point survey given to chiropractors, along with a summary of the OS notes.

#### Environmental scan

Environmental scans were conducted pre- and post-intervention to assess the following:

1. the number of staff, including chiropractors, at each clinic;
2. the number of examination rooms;
3. the presence and placement of the state quit program products and other cessation information (i.e., posters; brochures; palm cards, with the quitline telephone number for patients to self-enroll; foldover cards, wallet cards, and tear-off sheets);
4. the presence of indoor and outdoor smoke-free signage;
5. the presence of ashtrays or other receptacles used for tobacco products within 20 ft of all doors and operable windows; and
6. the presence of any written policies or guidelines related to the smoke-free policy or AAR.

#### Evidence of systematic processes in place to conduct AAR

To assess whether chiropractors had evidence of a *systematic process in place* for conducting AAR, including asking about use of electronic nicotine delivery systems (ENDS) and exposure to secondhand smoke, chart reviews were conducted. Data were collected at baseline four months retrospectively, at the AD visit described previously, and monthly for six months thereafter.

#### Evidence of AAR implementation and systems change

In addition to assessing whether a systematic process was in place, *implementation* of the AAR process was also evaluated through a chart review at baseline and monthly for six months thereafter to capture the following information per month for each chiropractor:

1. percentage of patients asked whether they used tobacco products (including ENDS) at the first visit for every NEC;
2. percentage of NEC patients identified as tobacco users advised to quit tobacco use; and
3. percentage of NEC patients identified as tobacco users referred for cessation services.

For documentation of baseline AAR rate, we reviewed NEC charts for the past four months. If electronic health records (EHRs) were used for charting, all charts were reviewed. If paper copies were used for charting, a systematic random sample of 20% of charts or 40 charts, whichever was more, for the past four months was reviewed. After the study began, we did the same review of charts once a month for the subsequent six months.

### Patient outcomes

Patient outcome measures, self-reported by those patients who were advised to quit or referred to quit, included the following:

1. type of tobacco used at NEC,
2. number of quit attempts,
3. use of evidence-based or other treatments to support quitting, and
4. successful quits, determined as point prevalence rates reported by patients on telephone interview at 30 days and three months after referral.
  Up to five attempts to reach the patient were made, if necessary.

### Statistical analysis

Because of small sample sizes, the results of the chart review and patient outcomes were summarized and reported using frequencies and percentages for qualitative outcomes and means and standard deviations for quantitative outcomes. All analysis was performed in Microsoft Excel.

## Results

### Participants

The final sample of chiropractors consisted of six practitioners (five men and one woman) from six practices at six clinics, with an average age of 41 years (range, 30 to 58 years). The chiropractors had been in practice for an average of 13 years (range, 3 to 33 years), and four routinely screened patients for tobacco use prior to the study. Three were solo practitioners, and three were in a group practice with a single partner. Two used only paper records, and four used a combination of paper and EHRs. Four described themselves as in a mixed style of practice, and two called themselves “evidence-based” practitioners. Finally, they had an average of 103 patients provided chiropractic care in their practice (range, 30 to 200 patients) per month, with an average of 222 patient visits per month. Compared with the population of chiropractors in the region, our study participants were more often males in small practices and more likely to be practicing in a mixed or evidence-based style. Because of the small number of participants, the study did not use a control group, and all six practitioners underwent training for systems change.

### Academic detailing

Each of the six clinics received AD from the OS staff, and all AD tasks were completed. Review of each clinic’s systems change workflow and implementation plan developed during the educational session was offered, and none felt the need to review. In a review of the forms currently being used by each clinic, two clinics had started process change prior to the first AD visit and received reinforcement, and four clinics received AD assistance and education. A system to prompt the chiropractor to conduct and document AAR was discussed, with none of the clinics having chosen a preferred method prior to the initial AD visit. Clinics then chose one of the following as a prompt: an electronic system (*n* = 2), a paper “sticky note” system (*n* = 2), use of the intake form (*n* = 1), and a flag/color system for patient charts (*n* = 1). For a method of referral, five clinics chose the fax referral option and one clinic chose an online method. Three clinics were also interested in using palm cards with the quitline telephone number for patients to self-enroll. Tracking of tobacco users and referrals was discussed, with clinics choosing to use electronic/diagnosis codes, dot stickers, a spreadsheet, and sticky notes, and keeping faxed referral forms on file.

Identification of a “clinic champion” was discussed, with clinics choosing one of the following: the chiropractor as the champion (*n* = 3), use of a team approach (*n* = 1), support staff as champion (*n* = 1), or no champion (*n* = 1). An ongoing internal feedback system was discussed, with some clinics interested in incorporating either a system for chart review for AAR performance (*n* = 1) or AAR follow-up tracking (*n* = 2). No clinics were interested in incorporating a formal policy for AAR. The process for ordering and identifying new educational materials was reviewed with additional staff educated on this process. Clinics designated who would be responsible for ordering materials, as follows: the chiropractor (*n* = 3), the chiropractor and support staff jointly (*n* = 2), or designated support staff (*n* = 1). The OS reviewed the sample job description for staff orientation in the educational materials.

Development of a tobacco-free workplace policy was discussed with each clinic. One clinic had a statement already in place in lieu of a formal policy, and two clinics showed interest in developing a policy.

Prior to the initial AD visit, five clinics had decided on a system for the *Ask* component, but no other components of the AAR system were in place until after the AD discussion with the OS. Plans for incorporation of the overall AAR workflow were discussed, with clinics determining that patients would either initially complete forms asking about tobacco use via the patient intake form (*n* = 5) or use a separate form specifically for tobacco use (*n* = 1); all clinics decided that the workflow would be the same for both new and established patients. Five clinics decided to perform the *Ask* component at the first patient visit, and one clinic decided to do this at the second visit, with *documentation* directly on the patient intake form (*n* = 4), by use of a sticky note system (n = 1), or use of both sticky notes and the intake form (n = 1). All clinics determined that the chiropractor would perform the *Advise* component in the examination room at the same visit that *Ask* was performed, with documentation through the use of an electronic prompt, sticky note system, spreadsheet/notes, formal AAR form, intake form, or EHR. It was also decided that the *Refer* component would be discussed with patients by chiropractors in the examination room, using the same documentation methods as *Advise*. The completion of referrals would also be performed by chiropractors on the same day as the discussion. Documentation of completed referrals included filing of the fax referral form and use of patient chart notes, spreadsheet/notes, the intake form, or the EHR.

At the end of the initial AD visit, the OS completed the SPS with all chiropractors (*N* = 6) participating. All chiropractors “engaged” with the Standard Patient before using AAR to address tobacco use, with the level of engagement ranging from a very quick style to building a caring foundation by briefly addressing the primary reason for the visit first. All chiropractors conducted AAR with the Standard Patient, with three chiropractors referring to the intake form during the *Ask*. component. The style of the *Advise* component was individualized, with chiropractors referencing the health concerns of tobacco (*n* = 4), including citing the connections among tobacco use, back pain, and healing (*n* = 2), and using MI techniques (*n* = 1). Two chiropractors advised using a more indirect approach (“I’m on your side and I know you know the harmful effects”) and a softened approach (“This may come as no surprise to you, as your health care provider, I recommend you quit using tobacco”). There was more commonality in the way chiropractors referred the Standard Patient, either by providing a brief explanation of the quitline program (*n* = 4) or by stating a plan to continue discussing tobacco use at their next visit (*n* = 1). The supportive behaviors assessed by the OS using the aforementioned six-point survey revealed a median score of six (“considerably”) for all components. The OS reported that of the six participants, only one was not as confident as the other chiropractors in the delivery of AAR.

### Environmental scan

Environmental scans were completed for all chiropractic offices (*N* = 6) pre- and post-intervention.

#### Number of staff and examination rooms

The number of clinic staff changed slightly between pre- and post-scan. Pre-intervention, the number ranged from two to three per clinic (mean = 2.67), with one clinic increasing staff members from three to four post-intervention (mea*n* = 2.83). The number of examination rooms changed slightly; pre-intervention, the number ranged from two to three (mea*n* = 2.83), with one clinic decreasing from three to two rooms (mean = 2.67) post-intervention.

#### State quit program products

Pre-intervention observations assessing the presence of the state quit program products found that none were present in any of the clinics. Post-intervention, four of the six clinics (66.7%) incorporated the state quit program products into their clinics, including posters (*n* = 4 clinics), brochures (*n* = 2 clinics), and tear-off sheets (*n* = 2 clinics). The following quitline products were not observed post-intervention in any clinic: palm cards, foldover cards, or wallet cards. None of the clinics incorporated other cessation, secondhand smoke, or ENDS educational or referral materials from the state quit program or other sources into their clinics.

#### Signage, ashtrays, and written policies/guidelines

Smoke-free signage was not present indoors in any of the clinics. Smoke-free signage on some or all of the doors into the buildings was present for three clinics pre- and post-intervention. Ashtrays were not present within 20 ft from any door or window of any clinic either pre- or post-intervention. Written policies or guidelines related to smoke-free clinics or AAR were not present in any clinic either pre- or post-intervention.

#### Evidence of systematic processes in place to conduct AAR

The baseline chart review was conducted in each of the six clinics. At baseline, two of the six clinics had evidence of a systematic method for the *Ask* component for NEC patients. No clinics had evidence of a systematic method for *Advise* or *Refer*, or to assess for ENDS use or exposure to secondhand smoke. By the end of the fourth month of the intervention period, processes were in place for AAR in all clinics, to assess ENDS use by 50% of the clinics, and to assess exposure to secondhand smoke by 67% of the clinics.

### Evidence of AAR implementation and systems change

See Table 1 for a summary of AAR implementation at baseline and over the six-month study period.

**Table 1:** AAR implementation by chiropractor

Chiropractor	Month	No. of NECs	% Asked	No. of users	% Advised	% Referred
1	Baseline	33	100.0	0	NA	NA
	1	7	100.0	0	NA	NA
	2	4	100.0	0	NA	NA
	3	8	100.0	1	0.0	0.0
	4	7	100.0	2	50.0	50.0
	5	5	100.0	0	NA	NA
	6	11	100.0	0	NA	NA
2	Baseline	24	0.0	NA	NA	NA
	1	12	83.3	7	100.0	71.4
	2	18	94.4	6	83.3	33.3
	3	18	100.0	2	100.0	100.0
	4	21	81.0	4	50.0	25.0
	5	21	95.2	4	75.0	75.0
	6	6	100.	1	0.0	0.0
3	Baseline	40	100.0	18	0.0	0.0
	1	33	100.0	5	0.0	0.0
	2	21	100.0	4	25.0	0.0
	3	20	100.0	2	50.0	0.0
	4	30	100.0	6	83.3	0.0
	5	40	100.0	6	16.7	0.0
	6	26	100.0	3	0.0	0.0
4	Baseline	39	100.0	17	0.0	0.0
	1	40	100.0	7	28.6	28.6
	2	25	100.0	2	50.0	50.0
	3	40	100.0	5	40.0	0.0
	4	39	100.0	2	0.0	0.0
	5	38	100.0	7	85.7	71.4
	6	38	100.0	8	37.5	25.0
5	Baseline	40	35.0	6	0.0	0.0
	1	29	37.9	8	75.0	0.0
	2	27	40.7	2	100.0	50.0
	3	10	100.0	0	NA	NA
	4	12	100.0	0	NA	NA
	5	9	100.0	3	0.0	0.0
	6	3	100.0	0	NA	NA
6	Baseline	35	100.0	6	0.0	0.0
	1	7	100.0	0	NA	NA
	2	15	100.0	0	NA	NA
	3	14	100.0	4	0.0	0.0
	4	16	100.0	1	0.0	0.0
	5	16	100.0	1	0.0	0.0
	6	22	100.0	1	0.0	0.0
All	Baseline	211	76.3	47	0.0	0.0
	1	128	84.4	27	55.6	25.9
	2	110	84.5	14	64.3	28.6
	3	110	100.0	14	35.7	14.3
	4	125	96.8	15	53.3	13.3
	5	129	99.2	21	47.6	38.1
	6	106	100.0	13	23.1	15.4

*NA* Not available

The performance in *Ask*, *Advise*, *Refer* (AAR) for each chiropractor individually and all chiropractors combined. For each month (including baseline), the following was recorded: the number of new episodes of care (No. of NECs), the percentage of NEC patients asked if they use tobacco (% Asked), the number of NEC patients identified as tobacco users (No. of users), the percentage of NEC patients identified as tobacco users advised (% Advised), and the percentage of NEC patients identified as tobacco users referred (% Referred)

#### Ask for NEC patients

At baseline, four of the six chiropractors asked all NEC patients whose charts were assessed whether they used tobacco; thus, these four chiropractors were not eligible for a systems change in *Ask*, although three of them developed a more comprehensive *Ask* component by adding questions to the intake forms. Of the remaining two chiropractors, at baseline, one chiropractor never asked about tobacco use, and one asked 35% of NEC patients. The chiropractor who never asked at baseline reached at least 50% at month 1 and remained at ≥50% throughout the study. The chiropractor who started at 35% for *Ask* surpassed 50% at month 3 and remained 100% thereafter. Therefore, successful systems change in *Ask* occurred in both applicable instances. It is noteworthy that all clinics asked patients at 100% of NECs by month 6. The number of NECs ranged from three to the maximum of 40 per clinic per month, with a mean of 19.7 (standard deviation [SD] = 11.7).

#### Advise for NEC patients

At baseline, no chiropractors advised NEC patients identified as tobacco users to quit tobacco use. The number of NEC patients who were tobacco users totaled 104 over the six-month intervention period and ranged from zero to eight per month for individual chiropractors, with a mean number of 2.89 (SD = 2.64) patients. Five of the six clinics succeeded in having some months of ≥50% of NEC patients who were tobacco users being advised, and three clinics achieved the formal definition (two consecutive months ≥50%) of systems change in *Advise*.

#### Refer for NEC patients

Referrals were included when documentation was present that indicated, either “indirectly” or “directly,” that an offer of a referral occurred. Indirectly meant documentation indicating that the patient was “not interested,” was already working with a primary care physician on quitting, was currently taking cessation medication, and so forth. Two clinics did not document referral of any NEC patients who were tobacco users; the chiropractor in one of these clinics indicated he only documented *Refer* if the patient was interested in quitting. Four clinics met the criterion of ≥50% of users referred for at least one month post-intervention; two clinics did so for one month, one clinic did so for two nonconsecutive months, and one clinic did so for three nonconsecutive months. None of the clinics achieved the formal definition of systems change in the *Refer* component.

### Patient outcomes: Direct patient follow-up of those interested in quitting

#### 30-day follow-up

Patients were given follow-up surveys via telephone at 30 days and 3 months post-referral. Among all patients advised or referred, 22 patients completed the consent form to be included in the follow-up surveys, and 15 tobacco users from two chiropractic clinics responded to the phone surveys, with 14 providing full responses (Table 2). The ages of the 14 respondents who provided demographic information ranged from 26 to 82 years, with a mean of 52.8 years (SD = 15.6 years). The majority of respondents were male (57.1%) and white (85.7%). All respondents completed high school or a GED. Only three respondents (21.4%) indicated they had insurance that assisted with tobacco cessation.

**Table 2:** Characteristics of tobacco users completing follow-up survey at 30 days and 3 months

Characteristic	30-day follow-up (*n* = 14)	3-month follow-up (*n* = 6)
Mean age ± SD, years	52.8 ± 15.6	55.0 ± 12.6
Gender, No. (%)		
Male	8 (57.1)	1 (16.7)
Female	6 (42.9)	5 (83.3)
Race, No. (%)		
White	12 (85.7)	5 (83.3)
American Indian/Alaska Native	2 (14.3)	1 (16.7)
Education, No. (%)		
High school/GED	3 (21.4)	2 (33.3)
Some college	3 (21.4)	1 (16.7)
Vocational/technical//trade school	3 (21.4)	1 (16.7)
Associate’s degree	2 (14.3)	0 (0.0)
Bachelor’s degree	3 (21.4)	2 (33.3)
Insurance assisting with tobacco cessation, No. (%)		
Yes	3 (21.4)	1 (16.7)
No	2 (14.3)	1 (16.7)
*DK/NS*	*9 (64.3)*	*4 (66.7)*

*DK* do not know, *NS* Not sure, *SD* Standard deviation

The majority of respondents (86.7%) indicated that they used cigarettes at the time of their NEC appointment, ranging from three to 30 cigarettes per day, with a mean of 14.6 cigarettes per day (SD = 8.9). Respondents also indicated use of cigars, cigarillos, or little cigars; chewing tobacco, snuff, or dip; a pipe; and ENDS.

All respondents indicated that they were advised to quit tobacco use during their chiropractic appointment, with 12 respondents (80.0%) recalling being referred to a specific quitline, class, or program. Fourteen respondents (93.3%) recalled being referred to the state quit programs. No patients recalled being referred to a local public health unit (LPHU) program or other resource.

Nine of the 15 respondents (60.0%) indicated that they had stopped use of tobacco for one day or longer since their NEC appointment because they were trying to quit for good, with two respondents (13.3%) indicating they had currently quit using all tobacco products for at least 30 days and five additional respondents (33.3%) indicating the intention to quit tobacco use in the near future. Of the nine respondents who had recently stopped using of tobacco for at least one day, three (33.3%) made a quit plan, two (22.2%) contacted the state quit program, and one (11.1%) contacted an LPHU to assist him/her with quitting. Only eight of these nine respondents answered questions regarding additional forms of assistance with quitting tobacco. One of these patients (12.5%) indicated receiving one-on-one counseling from a health professional, and two (25.0%) indicated using a medication to help them quit (Table 3).

**Table 3:** Patient survey responses regarding AAR, tobacco use, and quit assistance

Survey response	Answered “Yes,” No. (%)	
	30-day follow-up	3-month follow-up
AAR	(*n* = 15)	(*n* = 6)
Advised to quit	15 (100.0)	6 (100.0)
Referred to state quit program	14 (93.3)	6 (100.0)
Tobacco use at NEC	(n = 15)	(n = 6)
Used cigarettes	13 (86.7)	6 (100.0)
Used ENDS	3 (20.0)	0 (0)
Used other tobacco products	3 (20.0)	0 (0)
Currently use	(*n* = 15)	(*n* = 6)
Currently quit all tobacco products	2 (13.3)	1 (16.7)
Used tobacco in last 7 days	13 (86.7)	5 (83.3)
Used tobacco in last 30 days	13 (86.7)	5 (83.3)
Quit for one day or longer since NEC	9 (60.0)	4 (66.7)
	(*n* = 6)	(*n* = 1)
Intend to quit in near future	5 (83.3)	1 (100.0)
Quit assistance	(*n* = 9)	(*n* = 5)
Made a quit plan	3 (33.3)	3 (60.0)
Contacted state quit program	2 (22.2	3 (60.0)
Contacted LPHU	1 (11.1)	0 (0)
	(*n* = 8)	(n = 5)
Received one-on-one counseling	1 (12.5)	2 (40.0)
Used medication	2 (25.0)	0 (0)

*AAR* Ask, Advise, Refer; *ENDS* Electronic nicotine delivery systems; *LPHU* Local public health unit; *NEC* New episode of care

Results of the follow-up patient survey at 30 days and 3 months. Respondents included NEC patients identified as tobacco users. Implementation of AAR, tobacco use, and quit assistance was addressed. The number of respondents is provided in parentheses

#### Three-month follow-up

Of the 15 respondents who completed the 30-day follow-up, six completed a three-month follow-up survey (Table 2). During their 30-day follow-up survey, all six of these respondents indicated they used cigarettes but no other type of tobacco, were advised to quit all tobacco use, and were referred to the state quit program.

Four of these respondents (66.7%) indicated they had stopped using tobacco for one day or more since their NEC because they were trying to quit for good, with one respondent indicating he/she had quit all tobacco products for at least four months and one respondent indicating an intent to quit in the near future. Five respondents answered questions about methods or products they used to help quit tobacco. Three of these respondents (60.0%) indicated making a quit plan, three (60.0%) contacted the state quit programs, and two (40.0%) received one-on-one counseling from a health professional. None of these respondents contacted an LPHU, took a class, used an Internet or web-based program, used a cell-phone application, or used any medications (Table 3).

## Discussion

### Academic detailing

Each of the six clinics that received AD did not require review of their implementation plan, indicating their confidence in the training and education received prior to the initial AD visit. Chiropractors chose varying methods of prompting for, conducting, and documenting of AAR and for tracking referrals, indicating that implementation of AAR in the chiropractic setting needed to be unique to each clinic. The onsite AD was well received and assisted the clinics in the development and implementation of AAR.

### Environmental scan

Prior to the intervention, none of the chiropractic clinics had any cessation posters or other patient education materials present. By the end of the intervention, the majority of the chiropractors incorporated the state quit program’s products into their clinic environments. Although none of the clinics had smoke-free signage indoors, the legally required smoke-free signage on doors into clinics were present on some doors for half of the clinics, and ashtrays were not present within 20 ft of any clinic according to ND law (North Dakota Century Code § 23–12-10.4) [25]. None of the clinics incorporated written smoke-free policies or written policies related to AAR into their clinics by the end of the intervention period. Anecdotally, during the educational sessions, chiropractors remarked that it did not seem necessary to develop written policies for their practices because they were either sole practitioners or in a practice with one other chiropractor.

### Evidence of systematic processes in place to conduct AAR

Evidence that a systematic process to conduct AAR was in place was present in all clinics by the end of the fourth month of the intervention period. Additionally, systematic processes were in place to assess for ENDS use and exposure to secondhand smoke by 50% and 67% of the clinics, respectively.

### Evidence of AAR implementation and systems change

Although no clinic had sustained health system change for full AAR, clinics made progress in the individual AAR components. Per the study’s definition of systems change, only two clinics were eligible for systems change in the *Ask* component because the others were at 100% at baseline. These two clinics achieved systems change in *Ask*, and all clinics achieved 100% in *Ask* for all NECs by the sixth month. Furthermore, five clinics achieved sustained system change, as after systems change was achieved, the rate of tobacco user identifications did not drop below 50%. For the *Advise* component, five clinics succeeded in having individual months of ≥50% of tobacco users being advised, and three clinics achieved the formal definition of systems change, which is a change for two consecutive months. For the *Refer* component, no clinic achieved system change, although four had individual months of ≥50% of tobacco users being referred. Thus, for full AAR, four clinics successfully achieved this documentation of ≥50% patients for at least one month of the intervention period.

It is important to note that in other studies, successful health systems change was based on only the *Ask* component [18, 19]. Thus, our study went beyond these studies by requiring ≥50% for each AAR component for two months to be considered a health systems change. Also, at least one chiropractor in our study documented a referral as occurring only when a patient agreed to the referral rather than the offer of a referral. Future studies will need to refine more specific indicators and methods for the assessment and documentation for the *Refer* component.

### Patient outcomes

The quit rate was 13.3% (*n* = 15) for the 30-day follow-up and 16.7% (*n* = 6) for the three-month follow-up. The majority of respondents at both follow-ups had attempted to quit since their NEC appointment, and most of those who had not quit intended to in the near future. All respondents during both follow-ups indicated that they had been advised by their chiropractor to quit tobacco use, and 93.3% (n = 15) and 100% (n = 6) of respondents indicated being referred to the state quit program at the 30-day and 3-month follow-ups, respectively. Nationally, the rate of quit attempts for more than one day is 55.4%, and the rate for recent cessation for six months or more is 7.4%; the Healthy People 2020 goal for recent cessation is 8.0% [6, 26]. Although our study only assessed recent cessation for up to three months, our results provide evidence of the effectiveness of the intervention to implement AAR in chiropractic practices.

### Limitations

One limitation of the study is the self-selection of the chiropractors participating in this study and that they are not representative of the overall chiropractic population. A second limitation of this study is that there was only a small number of NEC patients who were tobacco users, and this may have affected the ability of chiropractors to gain experience in AAR to ultimately achieve systems change. A third limitation was the low response rate to the telephone survey by patients who were tobacco users.

It is important to note that major system changes in any medical model can take many years to be fully implemented and usually are hastened by the application of quality indicators or other obligations and incentives (e.g., financial) required by overseeing or accrediting bodies, which provides enticement for the system to implement the change. Thus, a six-month intervention period is a short time for systems change; additionally, some patients who were referred near the end of the study time frame could not be assessed for quit attempts or successful quits for 30 days or three months. A longer intervention period may have influenced the results.

Public health interventions are increasingly recognized as important to chiropractic practice, as illustrated by a recent white paper by the World Federation of Chiropractic [27]. With more than 30 million persons visiting a chiropractor each year in the USA alone [28], the potential for chiropractic to influence public health through tobacco interventions is huge. Thus, further study is needed, and we recommend a longer study time frame and continued education with AD for systems change to ensure that chiropractors and their patients continue on the trajectory seen in our study. Additionally, future studies are encouraged to include larger numbers of both practices and patients to improve response rates and reliability of results. Increasing participation by chiropractors may require collaborating earlier and more closely with national and state chiropractic organizations to encourage chiropractors to participate or by offering additional incentives for participation.

## Conclusions

This pilot study demonstrates the feasibility of implementing a systems change intervention in the chiropractic setting to identify tobacco users, to advise them to quit, and to refer them for cessation services. Chiropractors may need help in adapting the change to their unique practices and record-keeping systems. Use of cigarettes and other tobacco products continues to be a major contributor to morbidity and mortality, but chiropractors are well positioned to help their patients quit and improve their health. We encourage chiropractors and public health professionals to collaborate to implement systems change to improve population health.

## Abbreviations

AAR: *Ask, Advise, Refer*
AD: Academic detailing
EHRs: Electronic health records
ENDS: Electronic nicotine delivery system
LPHU: Local public health unit
MI: Motivational interviewing
ND: North Dakota
NDSBCE: North Dakota State Board of Chiropractic Examiners
NEC: New episode of care
OS: Outreach specialists
SD: Standard deviation
SPS: Standard patient scenario
The Guidelines: Clinical Practice Guideline: Treating Tobacco Use and Dependence: 2008 Update
